# Supplementary material for: Patient-Specific Circulating Tumor DNA for Monitoring Response to Menin Inhibitor Treatment in Preclinical Models of Infant Leukemia
Source: Cancers (Basel). 2024 Nov 28;16(23):3990. doi: 10.3390/cancers16233990 (PMC11640178; doi:10.3390/cancers16233990)
Supplement: Supplementary file 1 [file cancers-16-03990-s001.zip › cancers-3279581-supplementary.pdf]

Patient-specific Circulating Tumor DNA for Monitoring Response to Menin Inhibitor Treatment in Preclinical Models of Infant Leukemia

Louise Doculara[1], Kathryn Evans[1], J. Justin Gooding[2,3], Narges Bayat[1]*, Richard B. Lock[1]*

[1]Children's Cancer Institute, Lowy Cancer Research Centre, School of Clinical Medicine, UNSW Medicine & Health, UNSW Centre for Childhood Cancer Research, UNSW Sydney, Sydney, NSW, Australia

[2]School of Chemistry, UNSW Sydney, Sydney, NSW, Australia

[3]Australian Centre for NanoMedicine, UNSW Sydney, Sydney, NSW, Australia

*Authors contributed equally

SUPPLEMENTARY INFORMATION

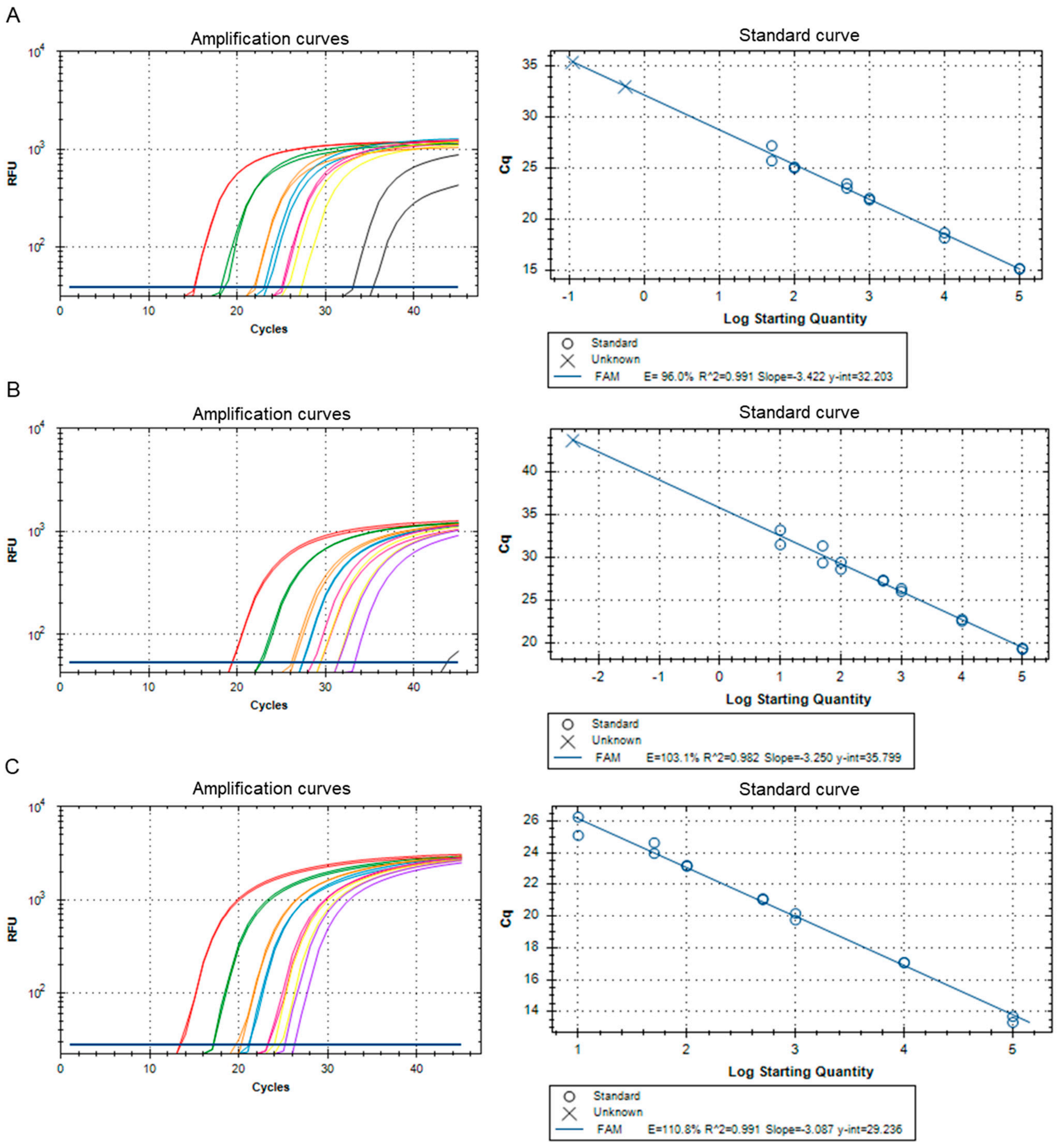

Supplementary Figure S1. Validation of PCR assays for ctDNA analysis of *KMT2A* fusions. Left panels, qPCR amplification plots; right panels, qPCR standard curves of serially diluted DNA extracted from MLL-r ALL PDX cell lysates in (A) MLL-1, (B) MLL-2 and (C) MLL-7. Ten-fold dilutions of 100 ng/µL DNA are shown: 10^{-1} (red), 10^{-2} (green), 10^{-3} (orange), $5x10^{-4}$ (blue), 10^{-4} (pink), $5x10^{-5}$ (yellow) and 10^{-5} (purple) were qPCR-amplified with the corresponding *KMT2A* fusion assay. Control samples: Peripheral blood mononuclear cells (PBMCs; black) from healthy volunteers. ALL-19 (*KMT2A* fusion negative) and non-template controls (NTCs) are not shown, indicating no amplification. Dark blue horizontal lines represent the negative threshold. RFU, relative fluorescence units. Two technical

replicates of each dilution are shown. Duplicates of ten-fold dilutions of 100 ng/µL DNA are shown from 10^{-1} to 10^{-5} (blue open circles). Line of best fit is shown. Cq, cycle; E, reaction efficiency; R^2, correlation coefficient; y-int, y-intercept.

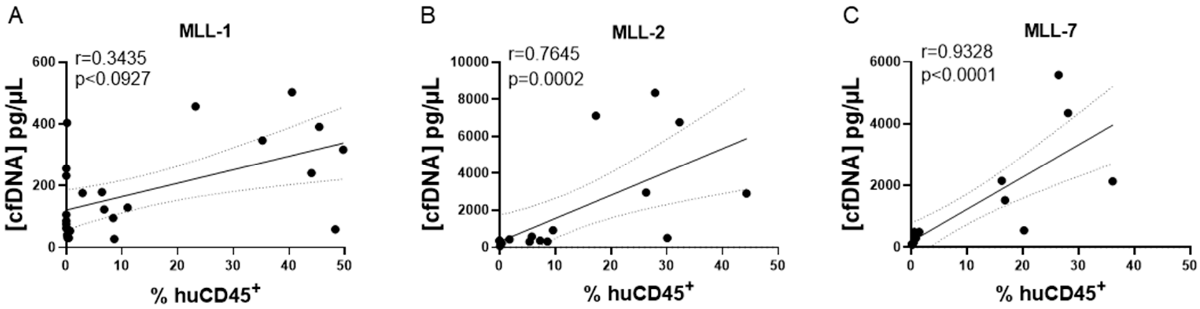

Supplementary Figure S2. Comparison of cfDNA and % huCD45$^+$ in NSG mice engrafted with MLL-r ALL PDXs. Spearman correlation analysis of cfDNA concentrations and % huCD45$^+$ in mice engrafted with (A) MLL-1 (B) MLL-2 and (C) MLL-7 PDXs at one-three weeks post inoculation. Dashed lines represent the 95% confidence limits of the best fit line. r = Spearman correlation coefficient; p = Spearman two-tailed correlation test.

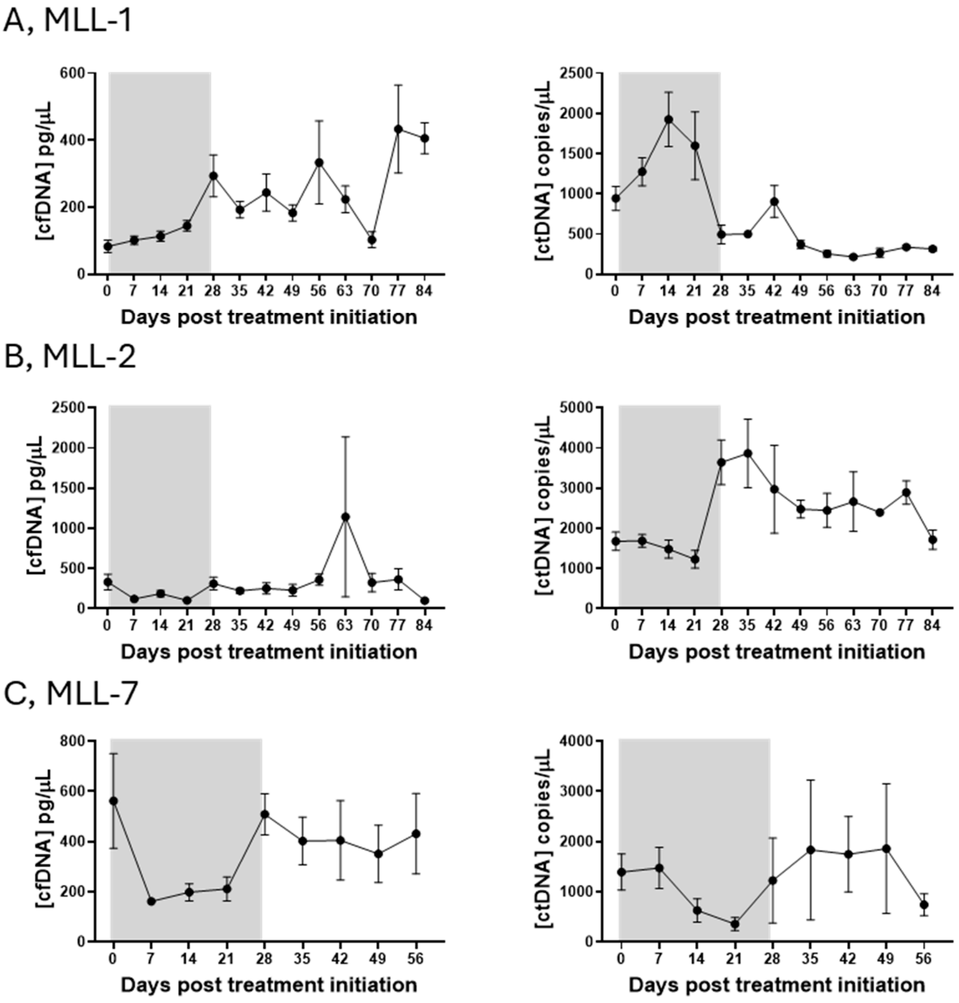

Supplementary Figure S3. Comparison of disease burden in the PB with plasma cfDNA and ctDNA concentrations in SNDX-50469-treated NSG mice engrafted with MLL-r ALL PDXs. Left panels, TapeStation analysis of cfDNA and right panels, ddPCR analysis of ctDNA at weekly timepoints from Day 0 (SNDX-50469 treatment initiation) in tail vein blood samples from 5-6 mice inoculated with (A) MLL-1, (B) MLL-2 and (C) MLL-7 PDXs. Data presented as mean values ± standard error of the means (SEM). The shaded grey area indicates the treatment window.

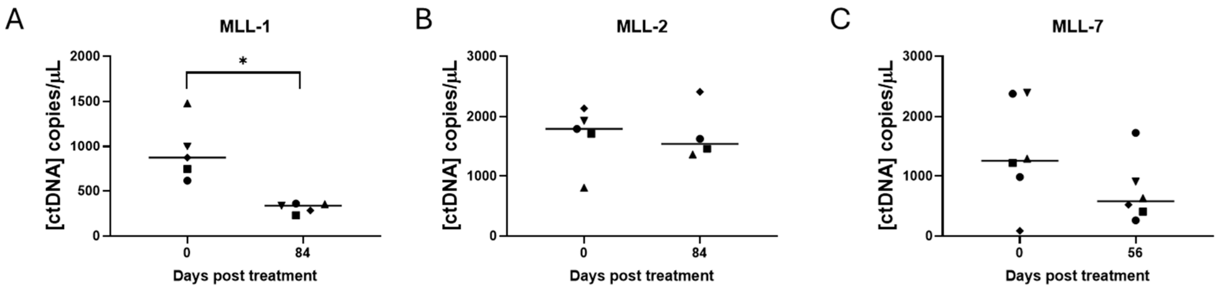

Supplementary Figure S4. Comparison of baseline ctDNA values from mice engrafted with MLL-r ALL PDXs with those at the end of the monitoring period post SNDX-50469 treatment. Comparison of ctDNA values obtained from mice engrafted with (A) MLL-1, (B) MLL-2 and (C) MLL-7 PDXs at the initiation of SNDX-50469 treatment (Day 0 baseline) and endpoint (unpaired t test with Welch's correction, * = $p < 0.05$, not significant for MLL-2 and MLL-7). Horizontal bar represents the median.

Supplementary Table S1. Summary of infant MLL-r ALL PDX responses to SNDX-50469. *p*-values were determined by the Gehan-Breslow-Wilcoxon test. EFS, event free survival; T-C, treatment minus control; ORM, objective response measure; CR, complete response (% huCD45$^+$ in PB <1% for at least 2 consecutively weekly readings during the study period); MCR, maintained complete response (% huCD45$^+$ in PB <1% for at least 3 consecutively weekly readings at any time after treatment has been completed).

	Treatment group	Median EFS (days)	EFS T-C (days)	EFS T/C (days)	Significance (*p*-value)	Median ORM
MLL-1	Vehicle	26.4				
	SNDX-50469	>90.0	>63.6	>11.9	0.0039	MCR
MLL-2	Vehicle	7.0				
	SNDX-50469	>90.0	>83.0	>12.8	0.0013	MCR
MLL-7	Vehicle	5.9				
	SNDX-50469	69.9	64.0	3.4	0.0013	CR
